# Erythropoietin prevents dementia in hemodialysis patients: a nationwide population-based study

**DOI:** 10.18632/aging.102227

**Published:** 2019-09-05

**Authors:** Peir-Haur Hung, Chih-Ching Yeh, Fung-Chang Sung, Chih-Yen Hsiao, Chih-Hsin Muo, Kuan-Yu Hung, Kuen-Jer Tsai

**Affiliations:** 1 Department of Internal Medicine, Ditmanson Medical Foundation Chia-yi Christian Hospital, Chia-yi, Taiwan; 2 Department of Applied Life Science and Health, Chia-Nan University of Pharmacy and Science, Tainan, Taiwan; 3 School of Public Health, College of Public Health, Taipei Medical University, Taipei, Taiwan; 4 Department of Public Health, China Medical University, Taichung, Taiwan; 5 Management Office for Health Data, China Medical University Hospital, Taichung, Taiwan; 6 Graduate Institute of Clinical Medical Science, School of Medicine, College of Medicine, China Medical University, Taichung, Taiwan; 7 Department of Internal Medicine, National Taiwan University Hospital, Hsin-Chu Branch, Hsin-Chu, Taiwan; 8 Institute of Clinical Medicine, College of Medicine, National Cheng Kung University, Tainan, Taiwan; 9 Center of Clinical Medicine, National Cheng Kung University Hospital, College of Medicine, National Cheng Kung University, Tainan, Taiwan

**Keywords:** Alzheimer’s disease, erythropoietin, end-stage renal disease, hemodialysis

## Abstract

Erythropoietic medications such as including erythropoietin (EPO) are known to be neuroprotective and to correlate with improved cognitive functions. However, it is not known whether supplementation with EPO reduces the risk of dementia in end-stage renal disease (ESRD) patients receiving hemodialysis (HD). Here, we determined whether EPO levels correlate with the incidence of different dementia subtypes, including Alzheimer’s disease (AD), vascular dementia (VaD), and unspecified dementia (UnD), and whether such associations vary with annual cumulatively defined daily doses (DDDs) of EPO for ESRD patients receiving HD. This retrospective study included data from 43,906 adult ESRD patients who received HD between 1999 and 2010. Using hazard ratios and Cox regression models, we found that patients receiving EPO had a 39% lower risk of general dementia than those in the non-EPO group. Similarly, the risks of VaD and UnD was lower for patients in the EPO cohort. The risk of dementia was further reduced in HD patients treated with EPO in combination with iron. Our results suggest that the use of EPO medications in HD patients is associated with a reduced risk of VaD and UnD, but not AD, regardless of whether EPO is used alone or in combination with iron.

## INTRODUCTION

In maintenance hemodialysis (HD) patients, renal anemia is generally treated with erythropoietic medications, including erythropoietin (EPO) and intravenous iron supplements. The introduction of EPOs has revolutionized the care of anemic patients with chronic kidney disease (CKD) and almost completely eradicated the severe anemia of end-stage renal disease (ESRD) patients. Moreover, EPOs decrease the need for recurrent blood transfusions and the risk of iron overload and may improve the patients’ quality of life. Today, EPOs and adjuvant iron therapy are the main treatments for anemia associated with CKD [1, 2].

Treatment of anemia with EPO was associated with improved neuropsychological test performance and electroencephalography measurements in uncontrolled studies of patients with ESRD conducted in the early 1990s [3, 4]. One study suggested that normalization of hemoglobin (Hb) using EPO was associated with further improvements in cognitive function [5], and other studies have suggested that EPO may exert neuroprotective effects independently of raising Hb levels [3, 4].

Due to its protective effects on cognitive function, EPO has been used in the treatment of neuropsychiatric disorders with cognitive impairments, including schizophrenia [6]. Given that the EPO receptor is widely expressed in the nervous system and that EPO easily crosses the intact blood–brain barrier [7], EPO supplementation is able to rescue cognitive decline in aged rats and restore impaired memory in vascular dementia (VaD) rat models [8, 9]. In addition, previous studies illustrated that EPO can attenuate hippocampal neuronal loss, neuroinflammation, and cholinergic deficit in rats [10], and can function as a neuroprotectant against amyloid beta (Aβ) toxicity [11, 12], which is a principal consideration for the development of treatments for Alzheimer’s disease (AD).

The data concerning the effects of EPO and intravenous iron supplementation on specific dementia subtypes among HD patients are scarce. Therefore, we conducted a total population-based retrospective cohort study to test whether EPO and intravenous iron supplementation correlate with the risk of various dementia subtypes including AD, VaD, and unspecified dementia (UnD) in HD patients.

## RESULTS

**Figure 1:**
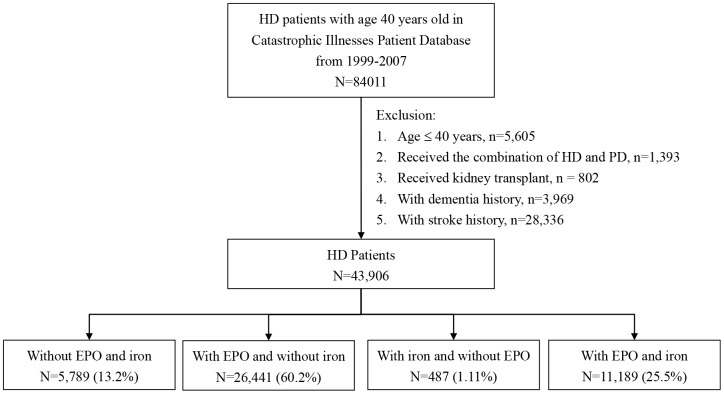
**Flow chart for classification of study subjects.**

The demographic characteristics and comorbidities of the HD patients in our cohorts are shown in Table 1. We recruited 43,906 HD patients who took EPO and 11,676 HD patients who took iron during the study periods. Of these, 11,189 patients in the EPO cohort (25.5%) and 487 patients in the non-EPO cohort (1.1%) were administered iron therapy for anemia (Figure 1). The most frequent comorbidities were hypertension (88.9%), anemia (51.6%), diabetes mellitus (51.2%), and hyperlipidemia (41.9%). Less than 5% of the study population had a history of atrial fibrillation (AF), and nearly 53.9% were aged 60 years or older.

**Table 1:** Demographic characteristics and comorbidities of hemodialysis cohort in Taiwan.

**Variable**	**Total**		**Without EPO and iron**		**With EPO and without iron**		**With iron and without EPO**		**With EPO and iron**		**p-value**
	**n**	**%**	**n**	**%**	**n**	**%**	**n**	**%**	**n**	**%**	
N	43,906		5,789		26,441		487		11,189		
Age (yr)											
Median (IQR)	62.4	(17.9)	56.5	(17.8)	62.5	(17.9)	63.6	(17.3)	60.6	(17.4)	<0.0001
41–50	8,336	19.0	840	14.5	4,934	18.7	79	16.2	2,483	22.2	
51–60	11,914	27.1	1,333	23.0	7,202	27.2	122	25.1	3,257	29.1	
61–70	12,437	28.3	1,711	29.6	7,453	28.2	154	31.6	3,119	27.9	
71–80	8,898	20.3	1,466	25.3	5,431	20.5	102	20.9	1,899	17.0	
≥81	2,321	5.29	439	7.58	1,421	5.37	30	6.16	431	3.85	
Gender											0.4555
Male	21,985	50.1	2,937	50.7	13,166	49.8	239	49.1	5,643	50.4	
Female	21,921	49.9	2,852	49.3	13,275	50.2	248	50.9	5,546	49.6	
Urbanization											<0.0001
Urban	24,142	55.0	3,111	53.7	14,291	54.1	360	73.9	6,380	57.0	
Suburban	14,437	32.9	1,954	33.8	8,870	33.6	97	19.9	3,516	31.4	
Rural	5,327	12.1	724	12.5	3,280	12.4	30	6.16	1,293	11.6	
Comorbidity											
Coronary heart disease	17,539	40.0	2,385	41.2	10,704	40.5	205	42.1	4,245	37.9	<0.0001
Hypertension	39,044	88.9	5,044	87.1	23,539	89.0	434	89.1	10,027	89.6	<0.0001
Diabetes	2,2465	51.2	3,180	54.9	13,480	51.0	270	55.4	5,535	49.5	<0.0001
Atrial fibrillation	618	1.41	89	1.54	382	1.44	7	1.44	140	1.25	0.4010
Heart failure	10,167	23.2	1,412	24.4	6,061	22.9	111	22.8	2,583	23.1	0.1197
Hyperlipidemia	18,384	41.9	2,169	37.5	11,124	42.1	216	44.4	4,875	43.6	<0.0001
Anemia	22,671	51.6	2,686	46.4	13,776	52.1	255	52.4	5,954	53.2	<0.0001
Annual DDDs, median (IQR)											
EPO (N=41,425)	140.6	(201.9)			116.8	(194.2)			192.8	(202.1)	
Iron (N=13,020)	8.60	(14.5)					6.64	(11.1)	8.70	(14.6)	
Days between index date and drug use, median (IQR)											
EPO (N=41,425)	8	(69)			11	(119)			5	(25)	
Iron (N=13,020)	132	(533.5)					170	(504)	130	(534)	
Follow-up years, mean (SD)	4.48	(3.14)	3.03	(2.65)	4.47	(3.14)	4.61	(3.19)	5.24	(3.13)	
Dementia	1,621	3.69	286	4.94	1,002	3.79	28	5.75	305	2.73	<0.0001
All-cause mortality	19,154	43.6	3,274	56.6	11,556	43.7	217	44.6	4,107	36.7	<0.0001

SD, standard deviation; DDDs, defined daily doses; IQR, interquartile range; EPO, erythropoietin.

### Annual cumulative exposure to EPO and iron

Biosimilar EPOs can be discriminated from the endogenous protein by slight differences that may include posttranslational modification; consequently, they may be similar—but not identical—to the originator EPOs [13]. A previous population-based study indicated that EPO consumption by HD patients was similar for biosimilar and originator EPOs [14]. Defined daily dose (DDD) is the assumed average maintenance dose per day for a drug used for its main indication in adults [15]. To investigate the effect of dose, and to avoid higher cumulative EPO doses with longer patient follow-up periods, the cumulative use of EPOs was calculated as total prescribed annual DDDs (*i.e.,* the same as total dispensed DDD under this system). Upon dementia diagnosis, the cumulative EPO dosage was recorded as the total of annual DDDs from drug initiation to the day before the diagnosis. Patients who took EPO at least once after the index date were defined as EPO users; the remaining patients were defined as non-EPO users. Similarly, patients who received intravenous iron at least once after the index date were defined as iron users; the remaining patients were defined as non-iron users. Upon dementia diagnosis, the cumulative iron dosage was recorded as the total annual DDDs from drug initiation to the day before the diagnosis.

Therefore, using the daily records of prescribed erythropoietic medications during the follow-up period, we categorized patients who had received erythropoietic medications as the treatment group and those who did not receive the drugs as the control group.

### Factors associated with dementia incidence

The results of our analysis examining the association between the use of EPO or intravenous iron and the risk of developing dementia are shown in Table 2. Stratified Cox proportional hazard regressions showed that the hazard ratio (HR) for dementia in HD patients who took EPO within the follow-up period was 0.48 [95% confidence interval (CI) 0.42 to 0.54; p<0.0001] in comparison with the HD patients in the non-EPO cohort. We further analyzed the HR value after adjusting the data for diabetes, hyperlipidemia, hypertension, anemia, coronary artery disease, heart failure, and AF. The HR value obtained was 0.61 (95% CI 0.54-0.70; p<0.0001) in HD patients who took EPO.

**Table 2:** Incidence, hazard ratios and interaction (between EPO and intravenous iron) for dementia among hemodialysis cohort treated with EPO or intravenous iron.

**Treatment**		**N**	**Event**	**PY**	**Rate^a^**	**Crude HR (95% CI)**	**P**	**Adjusted HR (95% CI)^b^**	**P**
**EPO (annual DDDs)**									
No		6,276	314	19,759	15.89	1.00		1.00	
Yes		37,630	1,307	176,901	7.39	0.48 (0.42-0.54)	<0.0001	0.61 (0.54-0.70)	<0.0001
Low (<71)		12,369	443	56,935	7.78	0.50 (0.43-0.58)	<0.0001	0.72 (0.62-0.84)	<0.0001
Median (71-200)		12,384	394	60,536	6.51	0.42 (0.39-0.49)	<0.0001	0.53 (0.46-0.62)	<0.0001
High (≥201)		12,877	470	59,431	7.91	0.51 (0.44-0.59)	<0.0001	0.62 (0.54-0.72)	<0.0001
**Iron (annual DDDs)**									
No		32,230	1,288	135,836	9.48	1.00		1.00	
Yes		11,676	333	60,824	5.47	0.59 (0.52-0.66)	<0.0001	0.75 (0.65-0.86)	<0.0001
Low (<5)		3,858	86	22,302	3.86	0.41 (0.33-0.52)	<0.0001	0.53 (0.41-0.70)	<0.0001
Median (5-13)		3,913	96	19,314	4.97	0.53 (0.43-0.65)	<0.0001	0.61 (0.49-0.75)	<0.0001
High (≥14)		3,905	151	19,208	7.86	0.84 (0.71-0.99)	0.0388	0.98 (0.82-1.17)	0.8213
**EPO**	**Iron**								
No	No	5,789	286	17,514	16.33	1.00		1.00	
Yes	No	26,441	1,002	118,322	8.47	0.53 (0.47-0.61)	<0.0001	0.68 (0.59-0.78)	<0.0001
No	Yes	487	28	2,245	12.47	0.78 (0.53-1.15)	0.2144	0.90 (0.60-1.34)	0.5931
Yes	Yes	11,189	305	58,579	5.21	0.33 (0.28-0.39)	<0.0001	0.49 (0.41-0.58)	<0.0001
						Interaction P=0.26			

^a^ Incidence rate, per 1000 person-years

^b^ Adjusted for days between index date and drug use, age, gender, urbanization level, diabetes, hypertension, coronary heart disease, heart failure, atrial fibrillation, hyperlipidemia, and anemia.

EPO, erythropoietin; CI, confidence interval; HR, hazard ratio; DDDs, defined daily doses; PY, person-years.

### Effects of EPO or iron intake on general risk of dementia

Table 2 also shows the association between the annual DDDs and the risk of dementia. Patients who exhibited higher annual DDDs of EPO exhibited a decreased risk of dementia (28%–47%). Analyzing the annual DDDs of EPO indicated that the low-, medium- and high-dose groups exhibited reduced dementia rates compared with the non-EPO cohort, suggesting a reduction of risk for annual various DDDs of EPO <200.

An additional analysis demonstrated an association between iron use and a reduced dementia risk (HR: 0.75; 95% CI, 0.65–0.86). In the analyses examining risks associated with different iron dosage, only low and medium doses of iron (<5 annual DDDs and 5–13 annual DDDs), but not high doses (≥14 annual DDDs), were associated with a reduced risk of dementia (39%–47%) in patients with HD using the non-iron cohort as a reference group.

### Association between combined EPO and iron intake and general risk of dementia

**Figure 2:**
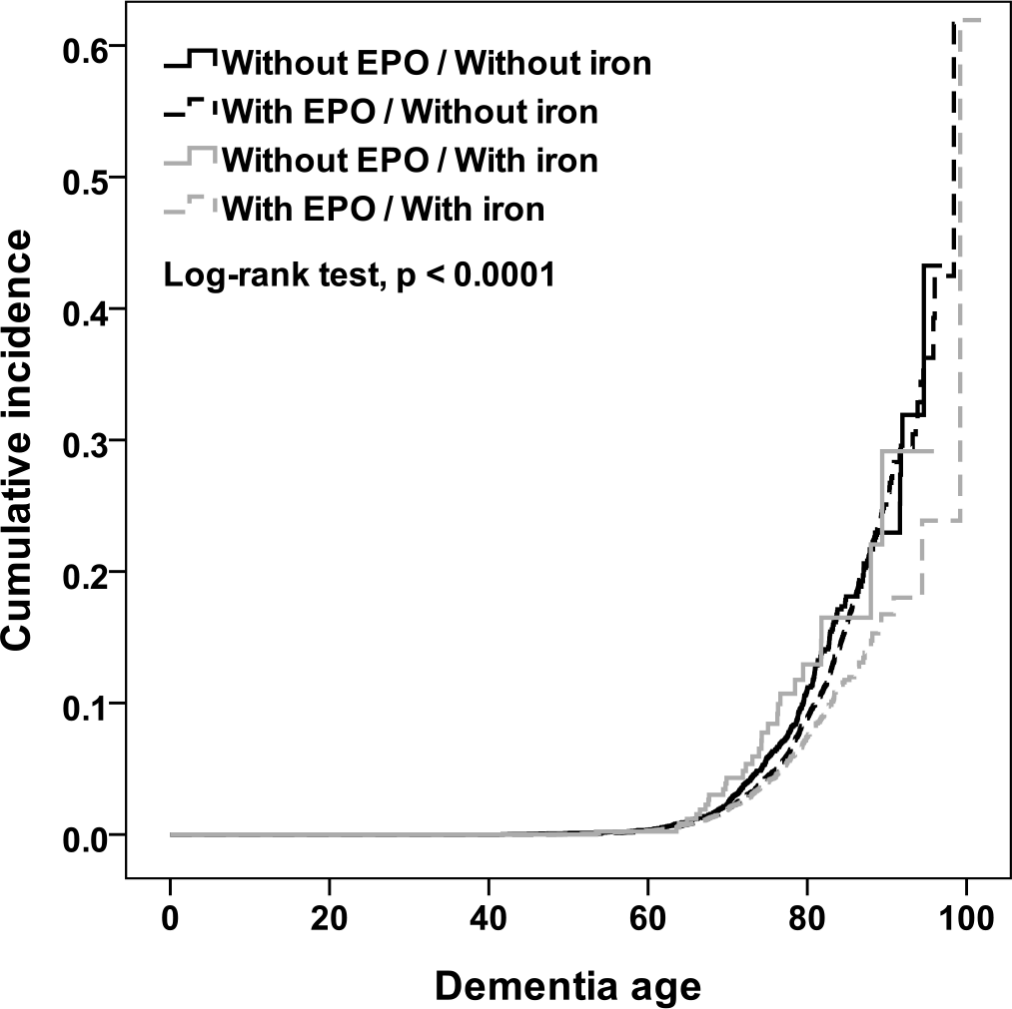
**Plot of cumulative probability of dementia incidence depending on dementia age among cohort patients who underwent different EPO and iron treatments.**

The risk of incident dementia in relation to the combination of EPO and iron use was evaluated in HD patients, as compared with those who used neither drug (Table 2). The Cox proportional hazards model revealed that after adjusting for age, sex, and comorbidities, the risk of incident dementia was reduced both among HD patients who had received both EPO and iron (adjusted HR = 0.49, 95% CI, 0.41–0.58), and those who had used EPO but not iron (adjusted HR = 0.68, 95% CI, 0.59–0.78). In addition, HD patients using EPO and iron treatments experienced a delayed onset of dementia or prevented it altogether (log-rank test, p < 0.0001, Figure 2).

### Association between EPO or iron intake and risk of dementia by dementia subtype

Table 3 shows the results of HR analysis for HD patient cohorts by dementia subtype. In comparison with non-EPO patients, HD patients treated with EPO were less likely to experience some subtypes of dementia during the follow-up period after the index healthcare use. Of note, the adjusted HRs for VaD and UnD in patients with HD were 0.44 (p<0.0001) and 0.65 (p<0.0001), respectively. Moreover, iron supplementation correlated with a reduced risk of UnD.

**Table 3:** Incidence, hazard ratios and interaction (between EPO and intravenous iron) for dementia subtypes among hemodialysis cohort treated with EPO or intravenous iron.

**Treatment**		**Alzheimer’s disease**					**Vascular dementia**					**Unspecified dementia**			
		**Event**	**Rate^a^**	**HR (95% CI)^b^**	***P***		**Event**	**Rate^a^**	**HR (95% CI)^b^**	***P***		**Event**	**Rate^a^**	**HR (95% CI)^b^**	***P***
EPO (annual DDDs)															
None		10	0.51	1.00			46	2.33	1.00			358	13.06	1.00	
Yes		36	0.20	0.65 (0.32–1.32)	0.2277		148	0.84	0.44 (0.31–0.62)	<0.0001		1,123	6.35	0.65 (0.56–0.74)	<0.0001
Low (<71)		7	0.12	0.48 (0.18–1.28)	0.1410		53	0.93	0.53 (0.35–0.80)	0.0027		383	6.73	0.77 (0.65–0.90)	0.0016
Median (71–200)		16	0.26	0.82 (0.37–1.84)	0.6349		39	0.64	0.34 (0.22–0.52)	<0.0001		339	5.60	0.56 (0.48–0.66)	<0.0001
High (≥201)		13	0.22	0.60 (0.26–1.37)	0.2214		56	0.94	0.49 (0.33–0.72)	0.0003		401	6.75	0.65 (0.55–0.76)	<0.0001
Iron (annual DDDs)															
None		34	0.25	1.00			152	1.12	1.00			1,102	8.11	1.00	
Yes		12	0.20	0.84 (0.38–1.84)	0.6561		42	0.69	0.78 (0.52–1.16)	0.2112		279	4.59	0.74 (0.64–0.87)	0.0002
Low (<5)		3	0.13	0.44 (0.09–2.07)	0.2994		14	0.63	0.81 (0.41–1.59)	0.5426		69	3.09	0.50 (0.37–0.68)	<0.0001
Median (5–13)		6	0.31	1.19 (0.46–3.07)	0.7145		12	0.62	0.66 (0.39–1.21)	0.1768		78	4.04	0.58 (0.45–0.74)	<0.0001
High (≥14)		3	0.16	0.69 (0.21–2.30)	0.5447		16	0.83	0.86 (0.51–1.46)	0.5836		132	6.87	1.01 (0.84–1.21)	0.9399
**EPO**	**Iron**														
No	No	10	0.57	1.00			39	2.23	1.00			237	13.53	1.00	
Yes	No	24	0.20	0.58 (0.27–1.23)	0.1552		113	0.96	0.52 (0.36–0.76)	0.0006		865	7.31	0.71 (0.61–0.82)	<0.0001
No	Yes	0	0.00	NA			7	3.12	1.60 (0.69–3.70)	0.2770		21	9.35	0.83 (0.52–1.31)	0.4127
Yes	Yes	12	0.20	0.48 (0.19–1.24)	0.1303		35	0.60	0.39 (0.24–0.64)	0.0002		258	4.40	0.50 (0.42–0.61)	<0.0001

^a^ Incidence rate, per 1000 person-years.

^b^ Adjusted for days between index date and drug use, age, gender, urbanization level, diabetes, hypertension, coronary heart disease, heart failure, atrial fibrillation, hyperlipidemia, and anemia.

EPO, erythropoietin; CI, confidence interval; HR, hazard ratio; DDDs, defined daily doses; PY, person-years.

The interaction between EPO and iron were 0.98, 0.07, and 0.52 in crude model, and 0.98, 0.10, and 0.52 in adjusted model for Alzheimer’s disease, vascular dementia, and unspecified dementia.

### Risk of dementia subtypes upon EPO or Iron supplementation

We estimated whether EPO supplementation correlated with reductions in risk for specific dementia subtypes based on the tertile of annual DDDs for EPO use. We observed a reduced risk of VaD (adjusted HR range from 0.34 to 0.53) and UnD (adjusted HR range from 0.56 to 0.77) among EPO users (Table 3).

Furthermore, the Cox proportional hazards model revealed a reduced risk of UnD among HD patients who underwent iron supplementation, adjusting for age, sex, and comorbidities (adjusted HR = 0.74, 95% CI, 0.64-0.87) (Table 3).

### Effect of combined EPO and iron supplementation on risk of dementia by dementia subtype

Table 3 illustrates the combined effect of EPO and iron supplementation on dementia subtypes. Compared with patients who did not consume EPO nor iron, patients who consumed EPO only presented a reduced risk of VaD (adjusted HR =0.52, 95% CI, 0.36–0.76) and UnD (adjusted HR = 0.71, 95% CI, 0.61–0.82). Similarly, patients who received both EPO and iron supplements concomitantly also presented a reduced risk of VaD (adjusted HR = 0.39, 95% CI, 0.24–0.64) and UnD (adjusted HR = 0.50, 95% CI, 0.42–0.61), but not AD (adjusted HR = 0.48, 95% CI = 0.19–1.24).

## DISCUSSION

Our total population-based retrospective cohort study revealed that long-term administration of EPO to HD patients was inversely associated with their general risk of developing dementia as well as specific dementia subtypes, including VaD and UnD. In addition, we also observed a reduced risk of dementia in HD patients supplementing with intravenous iron. To our knowledge, no prior study has explored the association between EPO supplementation, intravenous iron supplementation, and the risk of dementia subtypes.

Here, after controlling for potential confounders, we found that EPO use of less than 71 annual DDDs, 71–200 annual DDDs, and over 201 annual DDDs in cumulative dose is associated with a 28%, 47%, and 38% risk reduction in dementia, respectively, as compared with not using EPO. However, we found no consistent trends in risk reduction with EPO supplementation of 201 annual DDDs or greater. This paradoxical phenomenon could be attributed to the severity of medical comorbidities, or to a partial response to EPO. For example, patients who used higher annual DDDs very likely had higher resistance to EPO [16] or more medical comorbidities that could cause a poorer response. Indeed, 10%–20% of CKD patients with anemia are resistant to EPOs [17]. Despite their potential for neuroprotection, EPOs might not be sufficient to overcome the adverse effects of severe resistance to EPO [18] or other medical comorbidities. Our study was restricted to analyzing data for HD patients, who already suffer from a high risk of developing dementia; therefore, our results may not be applicable to non-HD patients and further research is need to characterize the effects of EPO supplementation among the general population.

The neuroprotective mechanisms of EPOs include decreased neuronal apoptosis, decreased inflammation, promotion of oligodendrocyte differentiation and maturation, and improved white matter survival [19–22]. Two randomized, double-blind studies assessed the effect of EPO treatment on cognitive function [6, 23]. They showed that intravenously-administered EPO leads to better performance in healthy subjects in a test of verbal fluency seven days after treatment [6, 23] and that weekly intravenous injections of EPO lead to improved performance in tests of cognitive function in chronic schizophrenic patients. However, large randomized trials to treat anemia in CKD or ESRD patients with EPO supplementation did not evaluate cognitive function [24]. Thus, there is currently insufficient evidence to justify changing current hemoglobin targets to prevent dementia in patients with CKD or ESRD. To our knowledge, our results here are the first to suggest that EPO supplementation correlates with a reduced risk of dementia and dementia subtypes in HD patients, regardless of whether EPO was used alone or combined with iron. Through our present study, after controlling for potential confounders, we also found that iron supplementation of less than 5 annual DDDs and 5-13 annual DDDs in cumulative dose is associated with a 47% and 39% risk reduction in dementia, respectively, as compared with no use of iron. Although EPOs have become the mainstay of anemia therapy in HD patients, iron deficiency and/or insufficient iron bioavailability emerges as a major limiting factor in the effectiveness of these treatments. Therefore, we conducted a subgroup analysis to study the effect of concomitant EPO and iron supplementation. We observed an adjusted HR of 0.49 (95%CI, 0.41–0.58) when compared with the no-EPO and no-iron subgroups. For many HD patients, intravenous administration of iron is often a prerequisite to elicit an optimal response to EPO.

The strengths of this study are its population-based survey with a large sample size, with good follow-up throughout. However, several limitations and precautions are needed for interpreting our results. First, some anemic patients can be asymptomatic and thus might not visit the clinic thereby eluding diagnosis; therefore, the incidence of EPO use in the nonanemic controls was probably overestimated because of the presence of these asymptomatic patients. In addition, unlike a previous study conducted by Kuo KL *et al.,* [25], which analyzed patient hemoglobin levels and medical records, the present study relied on anemia diagnoses by clinicians, which may be less sensitive and delayed. Second, our study was observational in nature and cannot prove causality. Although we adjusted for common health conditions, it’s possible that subclinical disease may also have contributed to cognitive decline. Third, hematological data such as ferritin and transferrin saturation (TSAT) were not available for us to consider them in the present study. Thus, it is not possible to evaluate whether iron was administered correctly or not. However, when it comes to prescribing EPOs to HD patients, physicians in Taiwan should follow the NHI reimbursement criteria to keep serum ferritin at levels >100 ng/mL and/or TSAT at levels > 20% during EPO therapy. We believe that the baseline iron parameters in our study might be in accordance with the NHI reimbursement criteria for all EPO users. Fourth, in the current study, the inclusion criteria of patients with dementia were strict, and only patients with at least three outpatient or inpatient claim records of dementia-related diagnosis codes were included; however, these criteria might still underestimate the number of patients with dementia, particularly among those who rarely visit hospitals or those diagnosed with dementia near the end of 2011. Fifth, patient hemoglobin or hematocrit levels were not available for us to consider them in the present study even though previous studies performed in Taiwan have shown that reasonable hemoglobin targets and favorable outcomes for CKD anemia can be achieved by intravenous iron supplementation [26–29]. Lastly, we did not analyze the effects of antihypertensive and oral hypoglycemic drugs, which may affect the progression of neurodegenerative diseases. Nonetheless, our study demonstrated a 47% risk reduction for developing dementia in HD patients who used EPO supplementation in the range of 71-200 annual DDDs. Moreover, our results suggest that intravenous iron supplementation correlates with lower risks of dementia in HD patients, especially in combination with EPO.

## METHODS

### Data collection

A universal National Health Insurance (NHI) program was implemented in Taiwan in March 1995. Ninety-six percent of the Taiwanese population has been enrolled in this program [30]. By the end of 1996, the Bureau of NHI (BNHI) had contracts with 97% of all Taiwanese hospitals and clinics to join the national health insurance system [31]. The NHRI safeguards the privacy and confidentiality of all beneficiaries and provides health insurance data for research only after ethical approval has been obtained. In this study, access to the National Health Insurance Research Database (NHIRD) was approved by the Chia-Yi Christian Hospital local Institutional Review Board (approval no. CYCH-IRB-2018069). Further research in different independent study cohorts could further test the correlation we have uncovered between EPO use and reduced risk of dementia.

### Study population

From the NHIRD database, we selected patients ≥ 40 years old who were beginning chronic HD treatment between January 1, 1999 and December 31, 2010 and who had survived more than 90 days of renal replacement therapy for ESRD (n=78,406). We excluded individuals younger than 40 years of age because their risk of dementia was negligible. ESRD patients are defined as those who had catastrophic illness registration cards for ESRD (International Classification of Diseases, 9^th^ revision, Clinical Modification [ICD-9-CM code 585]) and have started renal replacement therapy [32, 33]. We excluded ESRD patients who received the combination of HD and peritoneal dialysis (PD) (n=1,393) or who had undergone kidney transplantation (n=802) as well as those who had been diagnosed with incident dementia before their index clinic visits (ICD-9-CM codes from 290.0 to 290.4, 294.1, and 331.0 to 331.2) (n=3,969) or with any type of stroke (codes 430-438) diagnosed before or within 90 days of their index clinic visits (n=28,336). Thus, in the end we included a total of 43,906 incident HD patients in this study. For all individuals in the cohort we obtained data on potential confounders, which are documented risk factors for dementia [33], including hypertension (codes 401-405), diabetes (code 250), hyperlipidemia (code 272), coronary heart disease (codes 410-414), AF (code 427.31), anemia (code 280-285), and other forms of heart disease (codes 420-429), recorded during 12 months before their index clinic visits. Each patient was individually tracked from their index clinic visits to the end of 2011 to identify those who subsequently suffered from incident dementia. The date of any form of dementia diagnosis made for the first time during the follow-up period or by the end of the study was considered the study endpoint. In our study, types of dementia other than AD and VaD were categorized as UnD, such as frontotemporal dementia (FTD), Parkinson’s dementia, and dementia with Lewy body (DLB) or dementia of unknown etiology. Therefore, we grouped patients into AD (ICD-9-CM code 331.0), VaD (ICD-9-CM code 290.4), and UnD (ICD-9-CM codes 290.0-290.3, 294.1, 331.1, and 331.2) dementia subtypes [34].

### Statistical analysis

The primary endpoint of this study was to determine whether a patient had received ambulatory care visits or had hospitalizations for any type of dementia. We used Pearson’s χ^2^ test to compare EPO users and iron users with controls in terms of region of residence (urban, suburban, and rural) and selected comorbidities (hypertension, diabetes, hyperlipidemia, coronary heart disease, AF, anemia, and other forms of heart disease) at baseline. We considered these comorbidities only if the condition occurred in an inpatient setting or if there were two or more ambulatory care claims recorded one year before or after the index ambulatory care visit. EPO users and iron users were categorized into tertiles by annual DDDs to explore the potential effects on risk reduction. We measured dementia incidence using Kaplan-Meier analyses during the follow-up period for the HD patients with/without EPO or iron treatments.

The unadjusted HR along with the 95% CI was obtained by evaluating the association between HD patients with different drug usage and risk of dementia during the follow-up period using Cox proportional hazard regression. The adjusted HR was computed after adjusting for days between index date and drug use, age, gender, urbanization level, hypertension, diabetes, hyperlipidemia, coronary heart disease, AF, anemia, and heart failure. We further analyzed dementia incidence rates between cohorts according to dementia subtypes. All data analyses were conducted using the SAS (ver. 9.4) statistical package for Windows (SAS Institute, Cary, NC), and the significance level was set at 0.05 in a two-sided test.
